# Roadmap of integration of transgender health into healthcare systems: a TRANSCARE study

**DOI:** 10.3389/fpubh.2026.1894638

**Published:** 2026-07-13

**Authors:** Marilena Anastasaki, Agapi Angelaki, Filippos Paganis, Elena Olga Christidi, Nancy Papathanasiou, Christiana Gennata, Vasilis Kiosses, Eleni Panagiota Stoupa, Evika Karamagioli, Emmanouil Pikoulis, Christos Lionis

**Affiliations:** 1 Clinic of Social and Family Medicine, School of Medicine, University of Crete, Heraklion, Greece; 2 Health and Society Lab, School of Medicine and University of Crete Research Center, University of Crete, Rethymno, Greece; 3 Orlando LGBT+, Athens, Greece; 4 Post Graduate Programme of Global Health–Disaster Medicine, School of Medicine, National and Kapodistrian University of Athens, Athens, Greece

**Keywords:** equity, health, integration—and synthesis—oriented strategies, roadmap, transgender (GLBT) issues

## Abstract

In this study, we summarize evidence from the European project TRANSCARE to identify key areas for improvement in transgender care and synthesize our experience into a roadmap of knowledge integration into healthcare systems. According to our roadmap, as a public health concern, progress in transgender care requires its integration into health policy, education and training for healthcare professionals, and availability of essential gender-affirming medications and population-specific healthcare services, including both medical and social care. Our roadmap serves as a model for stakeholders to use when determining the elements and steps necessary for transgender care integration and includes the synthesis of multiple actions to address: (1) transgender care inclusion in national health policy, (2) legislation and finance, (3) availability of essential gender-affirming medications, particularly hormone therapy, (4) education and training for health care professionals, (5) implementation of trans-specific care services at primary and secondary care services, (6) implementation of trans-specific services in social care and linkage with medical care. Results and evidence from all project activities are combined to create a pathway with multiple nodes to guide the development of integrated transgender care and to monitor progress. While the study focuses on the health and social context of Greece, our roadmap serve as a model for adaptation in other settings. As with any public health issue, the implementation of the roadmap requires both stakeholder engagement and policy actions to be further piloted, tested, and eventually adopted.

## Introduction

Transgender people worldwide face substantial health disparities and barriers to accessing appropriate healthcare, including discriminatory healthcare services, lack of adequately trained providers, and obstructions by healthcare systems and insurance programs to cover trans-specific services (1). Discriminative attitudes among healthcare professionals and inherent heteronormativity in health services prevent all LGBTQI+ persons from accessing medical care. Furthermore, legal protection against discrimination in healthcare and other settings remain inadequate for this population in many European countries (2–6).

Specifically in Greece, attitudes toward the LGBT community are quite negative and discriminatory (7, 8). Greek LGBT organizations have been actively working to raise awareness about these issues in school education, along with targeted training of public officials, including teachers, police officers, and particularly healthcare professionals (9). Despite these developments, Greece shows high rates of discrimination and violence based on sexual orientation and gender identity (10–13). In 2014, the Greek parliament (N. 4285/2014) passed a law that prohibited hate crimes and hate speech related to gender identity and sexual orientation (among other fields). Two years later, the legal framework on equal treatment and combating discrimination (N. 4443/2016) was also updated to include prohibition of all forms of discrimination on the basis of gender identity (and sexual orientation) in the employment sector; however, this protection does not extend to the healthcare sector (14).

Although the law on legal gender recognition, which allows trans people to change their name and gender on official documents without the need for psychiatric evaluations or other medical procedures, was introduced in 2017 (L. 4491/2017), it has several shortcomings, including the costly and lengthy procedure that trans people must follow, the lack of gender options outside the binary, and the exclusion of married people and individuals under the age of 15. As a result, despite the current legal reform, not all trans people can easily access official documents that reflect their gender identity (14). Furthermore, although social health insurance in Greece covers hormone replacement therapy, it does not cover any other types of gender-affirming surgery. Individuals seeking medical transition procedures are still required to receive a psychiatric diagnosis of “gender dysphoria.” Additionally, although ICD-11 (which depathologizes trans identities while ensuring access to gender-affirming procedures) was issued in 2019 and was planned to come into effect in all countries by early 2022, it has not yet been effectively adopted in Greece (15).

Generic problems and contextual issues observed in the implementation of healthcare services in Greece, including the lack of integrated care and insufficient patient-centered approaches, further affect the delivery of appropriate care for transgender people (16, 17). Transgender care is not part of any medical curricula, sustaining the knowledge gap among healthcare professionals and the lack of country-specific guidance and resources on transgender health. Primary care has proven to provide an effective setting for designing and implementing novel approaches to enhance care for transgender individuals (18). However, even the recently launched Primary Healthcare Reform has not accounted for the integration of transgender health into the country’s health system (19).

The EU-funded program TRANSCARE (accessible at “https://transcare-project.eu/ as of 15 March 2024”) aimed to improve access and support the provision of quality care to transgender individuals. Several project results have been delivered during its 3-year duration, including a problem-mapping literature review, two large-scale needs assessment surveys, an online eLearning training course for healthcare professionals, numerous nation-wide information and awareness-raising activities, and a full-scale policy recommendation report. A recent scoping literature review of our working group highlighted that improving transgender care is a multidimensional issue that should be addressed at the societal, healthcare, and research levels, with future educational actions focusing on respecting transgender identity, protecting confidentiality, creating trusted provider-patient relationships, and providing sufficient competency on trans-specific healthcare issues (20). The integration of educational programs and health policy recommendations into the healthcare system is crucial to achieve community-integrated health, and several frameworks and methods have been proposed to guide the process and monitor the progress of care integration organizations by organizations, such as the WHO and other organizations (21).

By leveraging insights from all stakeholders, activities, and results of the TRANSCARE project, this report aims to identify key areas for improvement in transgender care and synthesize our experience into a roadmap of knowledge integration into healthcare systems.

## General approach

As a public health concern and according to our roadmap, progress in transgender care requires integration into health policy, education and training of healthcare professionals, availability of essential gender-affirming medications and population-specific healthcare services, including medical as well as social care. The TRANSCARE Integration Roadmap (TIR) serves as a model for stakeholders to use in determining the necessary elements and steps for transgender care integration. According to WHO’s example of roadmap creation (21), the TIR includes the synthesis of multiple actions to address:

1) transgender care inclusion in national health policy
2) legislation and finance
3) availability of essential gender-affirming medications, particularly hormone therapy
4) education and training of health care professionals
5) implementation of trans-specific care services at primary and secondary care services
6) implementation of trans-specific services in social care services and linkage with medical care

**Figure 1:**
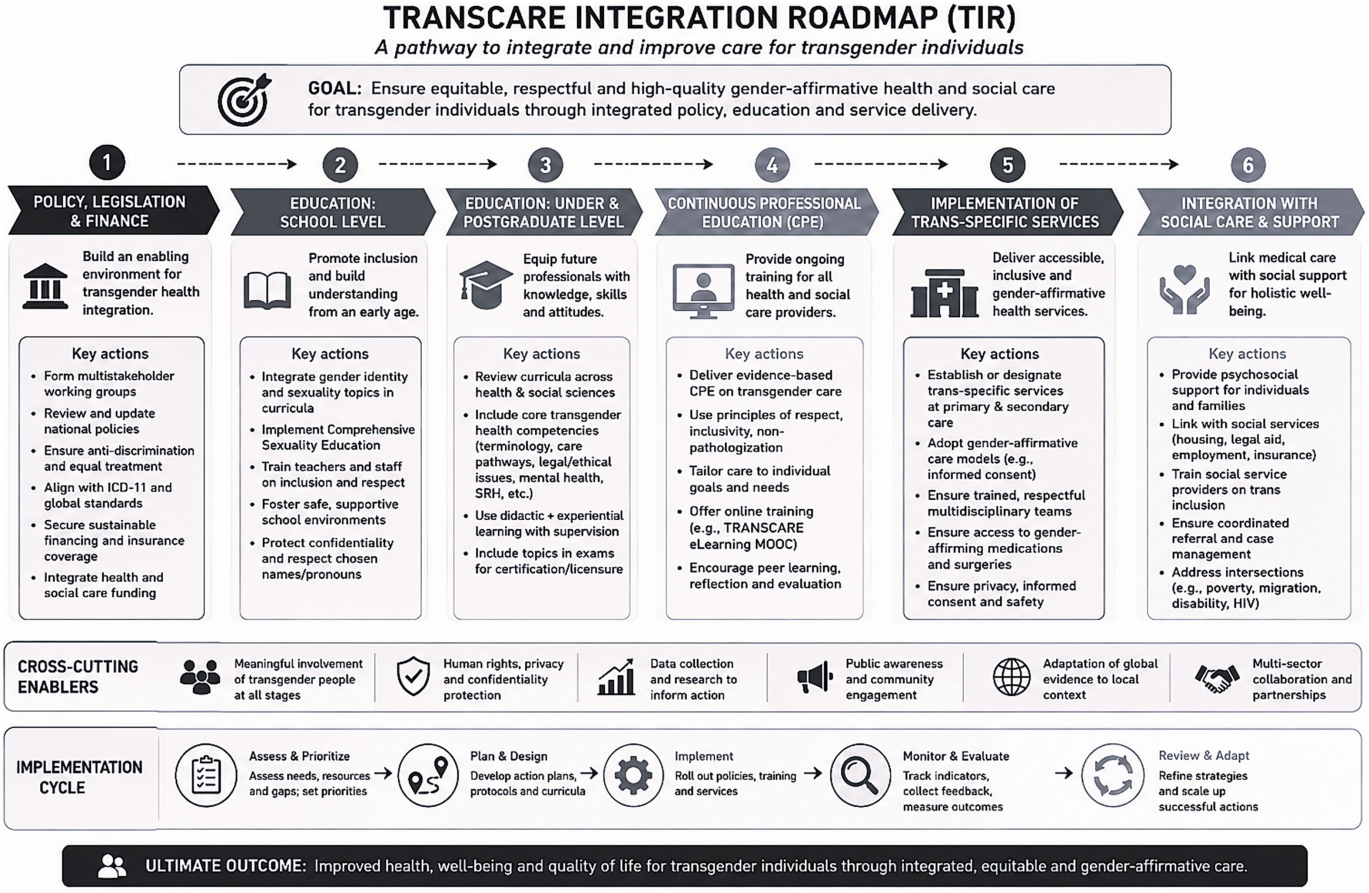
The TRANSCARE roadmap for transgender health integration.

As shown in Figure 1 and explained in detail in the subsequent sections, results, and evidence from TRANSCARE work packages were drawn to create a pathway with multiple nodes to guide the development of integrated transgender care and monitor respective progress. The project focuses on the health and social context of Greece; however, our roadmap could be used as an example for adaptation in other settings as well. As with any public health issue, implementing the TIR requires both stakeholder engagement and policy actions to pilot, test, and eventually adopt it.

## Policy, legislation, and finance

As part of the TRANSCARE project, two Policy Roundtables were held under the coordination of the Medical Association of Athens, engaging representatives from public healthcare institutions and civil society organizations, with the aim of developing policy proposals to improve trans people’s access to healthcare services in Greece. Among the key points that emerged from the round table discussions and should be addressed by policy, legislation, and finance activities to integrate transgender health into the healthcare system were:

- The need for a multifaceted strategy to guarantee trans people’s access to health services
- The need to address discrimination based on gender identity and expression through institutional and practical interventions
- Trans people’s active involvement in the planning and execution of interventions should be guaranteed
- The need to formulate policies to ensure equal treatment of transgender individuals at all levels. Specifically, the need to address the systemic and educational gaps that hinder trans people’s access to healthcare, such as misgendering and deadnaming by healthcare providers, problematic diagnoses and pathologization, and obstacles in obtaining prescriptions
- The need to tackle the absence of insurance funds for gender-transition procedures, the limited availability of trans-specific healthcare services resulting in appointment overload and delays, and a shortage of specialized healthcare professionals. Consequently, the need to establish specialized clinics for gender reassignment counseling, following the standards set by exemplary countries, was acknowledged
- The immediate need to upgrade the entire healthcare system to align with the World Health Organization’s new classification system, ICD-11, and the necessary adaptation procedures for accessing gender reassignment medical services

According to the WHO’s Public Health Model (21), when creating a roadmap for integrating transgender health into the healthcare system, it is essential to establish specific working groups to elaborate on the aforementioned needs. These groups should consist of national experts on transgender health, international consultants, representatives of the trans community, and representatives from ministries and organizations responsible for health policy. Existing national health policies should be reviewed to set priorities based on the identified needs, approve additions and updates, and subsequently include transgender health in all health policies and documentation, including (among others) trans-specific areas of focus such as non-communicable diseases, HIV, sexually transmitted and other infectious diseases, mental health, and gender affirming medication policies.

Legal expert groups should be further formed, including the above stakeholders as well as legal, ethical, and human rights experts. Their role is to review the legal components of health and drug policy, finance, and the frameworks of transgender health integration, including, for example, potentially special licensing for establishing essential trans-specific facilities, registrations as medical services, and workforce allocation (21).

Finally, a finance expert group with additional contributions from health economists, health system managers, and health as well as social service providers is crucial for reviewing existing funding and service delivery models and determining the cost-effectiveness of delivering transgender health services, including primary and secondary care as well as social care. Such expenses may include costs of establishing new facilities, salaries of personnel medication, equipment, and other direct and indirect costs (21).

All the above expert groups in collaboration should further address issues related to insurance coverage for trans-specific care and medical procedures, taking into consideration the socio-economic determinants of health for the transgender population. Another important area that requires collaborative work concerns the integration of health and social care services and, importantly, who is responsible for their funding, since in many countries such as Greece, health and social care are funded by different Ministries. Other organizational issues, including salaries, additional compensations for professionals providing out-of-hours trans-specific services, and availability for external funding to promote transgender health, should also fall into the agenda of policy, legal, and finance elaboration (21).

## Education

TRANSCARE has produced extensive results to identify and address the educational needs of healthcare professionals and the public on transgender health. Based on a literature review, observational studies among healthcare providers, medical students, and trans individuals, and two policy round tables, the TRANSCARE group has highlighted that transgender health should be integrated into all levels of education, namely:

- School education
- Under- and post-graduate education in medicine and other health and social sciences
- Continuous medical education of health and social care providers

Detailed information on necessities and actions that need to be taken at each level is summarized below:

### School education

The results of the two TRASCARE and policy round tables acknowledged the necessity and intention to revamp the primary and secondary education systems regarding sexuality education by incorporating topics on gender identity and sexuality, as well as transgender identities. Importantly, including Comprehensive Sexuality Education courses, which are currently entirely lacking and merely resisted at all school levels and university curricula in Greece and many other countries, was highlighted as an immense need. Based on the results of the TRASCARE field research, there seems to be an agreement regarding the need for education that will cover, among others, topics including basic concepts related to gender identity, sexual orientation, and sex characteristics, terminology, and inclusive, non-stigmatizing communication and information concerning medical transitioning. Ensuring the staffing of educational units with well-informed and educated professionals who respect the identities of transgender and also intersex students, protect confidentiality, and establish safe and trustworthy relationships and school environments is also crucial.

### Under- and post-graduate education

In the framework of TRANSCARE, a mixed-methods study including an online survey and qualitative research focus group discussions was conducted to assess awareness, attitudes, perceptions, and training needs of health and psychosocial care professionals, as well as students of relevant faculties on gender identity, gender expression, and trans identities, as well as to document trans people’s own experiences from healthcare services. Based on the results of this research among 259 students from schools of medicine, nursing, psychology, and social work, the following needs were identified for the integration of transgender health in under- and post-graduate curricula:

- The need to clarify information on terminology, gender identity, and sexual orientation issues, as approximately 25% of students believe that trans identities are a choice. Additionally, clear references to trans issues in lessons are never (48%) or rarely (30%) made, and references to gender identity issues in educational textbooks are absent (43%) or rare (27%), while, in cases where they do exist, 25% said that they are pathologizing.
- The need to provide explicit training, as 86% of students agreed or strongly agreed that the staff at healthcare services is not adequately trained to provide services to trans people, while 63% of students reported that they had not received any kind of training outside the context of their university. Students also know little (38%) or not at all (44%) about the process a person needs to follow to start hormone therapy, and, respectively, little (33%) or not at all (48%) know about the process required for surgery.
- Currently, only two under-graduate programs in Greece include courses that specialize in LGBTI+ identities, both courses are elective.

The TRANSCARE policy round tables further identified the following:

- The provision of inclusive services to trans people depends on educating health professionals at all levels and increasing awareness of the issue.
- The need for education that will cover topics including gender identity and sexual orientation, terminology and inclusive, non-stigmatizing communication, transition procedures (medicines, services, insurance coverage, protocols, preparation and support of individuals), specialized healthcare needs, protocols, guidelines and best practices in Greek, contemporary scientific data, mental health focus, sexual and reproductive issues, legal framework, prevention and combating of discrimination in the healthcare sector update of medical records and registration forms and family support.

According to WHO’s Public Health Model (21) in creating a roadmap of integration of transgender health into the educational system of under and post graduate studies expert groups composed of deans of medical, nursing, psychology, pharmacy, and social work schools, education experts national and international transgender health experts and representatives of the trans community should be formulated to review existing curricula and adapt evidence-based examples from other countries into the local context. The aforementioned core transgender health competencies should be integrated into such curricula, and education must be both didactic and experiential with peer-to-peer learning and appropriate supervision. To facilitate the uptake of transgender health, respective themes should be included in course examinations required for certification and licensure.

### Continuous professional education and development

Identifying the educational needs of healthcare providers in order to develop an evidence-based and context-driven training module to improve access of transgender individuals to health care has been the cornerstone of the TRANSCARE project. Barriers and facilitators to care for transgender individuals were identified in a scoping literature review of TRANSCARE (20) and were later translated into educational recommendations through the work of the TRANSCARE policy roundtables. According to these, continuous professional education programs should be based (among others) on the following principles:

- Promoting empowerment and inclusivity, reducing stigma, and facilitating access to suitable healthcare for all individuals
- Respecting diversity and all gender identities, while avoiding pathologization
- Involving transgender and gender diverse individuals in the development and implementation of healthcare services
- Familiarizing with social, cultural, economic, and legal factors that may impact the health of transgender individuals, as well as their willingness and ability to access services
- Providing healthcare services (or referring to knowledgeable professionals) that affirm gender identities and expressions
- Tailoring treatment approaches to meet the specific needs of patients, taking into consideration their goals for gender identity and expression
- Focusing on promoting overall health and wellbeing and embracing harm reduction approaches when appropriate.
- Ensuring transgender and gender diverse individuals have complete and ongoing informed participation in decisions regarding their health and wellbeing
- Supporting and advocating for patients within their families and communities when appropriate

Based on the above, a Massive Online Open Course educational program for healthcare professionals was developed by the TRANSCARE consortium to address the identified educational needs and promote transgender health and access to services “https://e-learning.transcare-project.eu/ (Accessed March 15, 2024).” The education program is structured into broad teaching units (modules), each of which incorporates specific lessons. Each lesson may also include individual lectures (units). In summary, the program covers the following topics:

- Basic concepts and terminology: concepts (sex characteristics, gender expression, gender identity, sexual orientation, terminology), gender transition (social, legal, medical), and the transgender community in Greece (discrimination, invisibility, exclusion, hate crimes).
- Access to services—challenges and consequences: (De)pathologizing of LGBTQI+ identities (history and current state), challenges (healthcare system shortcomings, professionals’ attitudes/perceptions, training needs, access to gender transition services), legal framework (recognition, discrimination, hate crimes/speech, healthcare ethics codes, transgender rights), and personal reflection (self-assessment).
- Creating inclusive health services: inclusive structures (inclusion and accessibility, information recording, use of spaces, visibility enhancement, best practices), communication (patient-centered care, communication skills, non-verbal communication, trust/empathy, interaction), inclusive services (reception, medical history intake, examinations, collaboration/information sharing with colleagues, guidelines), and interconnection (importance of collaboration with LGBTQI+ organizations and specialized services, recording incidents of violence/discrimination, support, initiatives).
- Specialized services: mental health (minority stress, impact of discrimination, affirmative approach, transgender children and adolescents, guidelines), medical gender-affirming procedures (hormone therapy, surgeries, ICD-11, and access to services), sexual and reproductive health (trans individuals’ needs, safe practices, reproductive issues and parenting choices, guidelines), and intersectionality (definitions, challenges, best practices).

This was the first training program for healthcare professionals addressing the care delivery and management of transgender individuals, created in Greece through an ongoing process of collaboration, production, and feedback. The design of the program as a Massive Open Online Course (MOOC) was yet another innovation, with documented effectiveness in providing high-quality continuous education to healthcare professionals (22). The development and use of such programs, specifically designed for care delivery for transgender individuals, are very limited internationally. However, individual studies highlight professionals’ interest and the urgent need for adequate training in this specific area (23). The structure and tools of this program address the immediate need for the creation of inclusive services and trained professionals with the aim of enhancing transgender individuals’ access to healthcare and providing appropriate and high-quality services to this population. Ensuring the wide dissemination across professional networks and inclusion of this module in university curricula is important for the integration of transgender health into the educational and healthcare system of Greece and other countries.

## Practice implementation and integration

When it comes to the implementation and integration of transgender health into medical and social care systems, gender affirmation is a key to addressing the needs of the population and a unique social determinant of health that affects trans people’s lives (24). Gender affirmation refers to the process of being affirmed in one’s gender identity and is comprised of at least four constructs: social (chosen name and pronoun), psychological (respecting one’s gender, resisting stigma and transphobia), medical (hormone therapy, surgery), and legal (name change and gender marker change) (25). Gender-affirmative health care refers to care that holistically addresses transgender people’s physical, mental, and social health needs while respectfully affirming their gender identity (24). In this direction, the TRANSCARE policy round tables have further resulted in the following recommendations that health environments and professionals should take into consideration in order to deliver gender affirming care:

- Ensuring respectful and humble communication with trans patients in order to achieve positive outcomes in consultations. These include acknowledging that not all trans people are the same, understanding that it is inappropriate to ask personal questions about their transitioning process or genitals unless willingly shared or medically necessary, avoiding assumptions about their sexual history based on their gender identity, providing an environment where support persons can accompany the patient, using simple language and encouraging questions, and involving patients in the decision-making process through informed consent.
- Creating a sensitive, safe, and inclusive environment to facilitate more effective care for trans individuals. This includes intake and sign-in forms adapted to allow the use of chosen names and gender markers, offering an option for preferred/used names by asking trans patients directly and respecting their preferences, and ensuring safe access to gender-segregated spaces (bathrooms, wards) or offering gender-neutral options to ensure the comfort and safety of transgender individuals.
- Safeguarding privacy and confidentiality while fighting discrimination and stigma. These include adhering to strict privacy and confidentiality protocols, treating sex assigned at birth and trans status as confidential clinical information that is not accessible to administrative staff, and ensuring that gender history and status remain confidential at all times.

The following country-specific needs were further elaborated on in order to highlight the immense need for a multidisciplinary approach linking clinical care with mental and social services:

- The need to combat the unethical and abusive practices that transgender people, especially minors, encounter when seeking and receiving psychiatric treatment in Greece, agreeing that professional bodies such as the Hellenic Psychiatric Association should take measures to ensure the appropriate training of their members in response to this issue.
- The need to train social structures to offer transgender people and their families inclusive support services.

In addition, several actions, mainly implemented in the USA, that develop and deliver models of gender-affirmative care can serve as an example for integrating transgender health into the systems of Greece and other countries, as long as this integration is supported by policy will and incorporated into any organization’s culture. Such models include, among others:

- The Informed Consent Model, which comprises a powerful tool in gender-affirmative care, allowing trans individuals to make decisions regarding their readiness for gender-affirming medical treatments. This model is based on trans people’s self-determination and knowledge of their own needs and identities. It involves facilitating informed decision-making by providing access to information, discussing benefits and risks, and respecting the patient’s autonomy (26).
- The Fenway Health model offers accessible, patient-centered, gender-affirmative care for transgender individuals where gender affirmation (e.g., hormone therapy) is a routine part of primary care service delivery. Using an informed consent model of care, the model removes unnecessary barriers to hormone therapy for trans patients, including prolonged mental health evaluations to obtain hormone therapy, which had long been embedded in existing standards of care. Trans patients complete a hormone readiness assessment, but mental health counseling is not automatically required (27).
- The Callen-Lorde Community Health Center model provides quality and sensitive medical and related services, including trans-inclusive registration forms, gender-neutral bathrooms, and trans-affirmative signage and imagery, and trans-identified staff. The model early adopted a customized electronic health record that allows appropriate documentation of identity and anatomy among transgender patients. Also in this model, transgender health is embedded in primary care, with primary care professionals offering comprehensive transgender health care, including hormone therapy, using the Informed Consent Model that removes unnecessary restrictions to hormone access. The center has onsite care coordination and legal services to assist with name changes, housing, and insurance navigation, addressing structural barriers to care. The center provides low-cost or free hormones and education programs aiming to increase provider knowledge and clinical skills caring for transgender communities (28).

## Conclusion

The TIR presented in this synthesis suggests that care systems and clinical settings should consider actions that integrate social, psychological, medical, and legal components for the development of transgender care. Holistic and culturally responsive transgender health must be tailored to the needs of transgender communities, while addressing structural factors, namely stigma and transphobia in health care settings, is necessary to increase uptake of the full care continuum (e.g., prevention, diagnosis, treatment, and linkage to care) for the transgender population. Training of health care providers and improving systems of care delivery to be gender-affirming are integral components of any strategy to improve accessibility of transgender individuals to care. Involving transgender people in all aspects of care and working with transgender communities while forming meaningful engagement of local, national, and global transgender communities is paramount to ensure responsiveness of interventions and programs, as well as to increase trust and reciprocity between clinicians, researchers, patients, and participants.

Our roadmap identifies key tasks and obstacles that must be overcome for transgender care to be seriously included in the Greek healthcare agenda and primary care reform, which is currently unfolding (19) but can also be used to adapt respective actions in other countries. This is particularly relevant as transgender health is still underdeveloped worldwide, and not many countries have key policies for transgender care to be accepted. Access to hormone therapy is still too difficult in many countries, a limited proportion of the healthcare workforce has received trans-specific education, and too few services are currently operating to meet the population’s needs. As with any health issue, the key to the integration of transgender care is the political will to reallocate care resources and to better manage health care costs.

## Group member of the TRANSCARE group

Pikoulis E., Karamagioli E., Stoupa E, Lionis C., Anastasaki M., Angelaki A., Panagiotopoulou A., Kaouni P., Linardis N., Dedes N., Kokkali E., Kouris G., Christidi O., Paganis F., Papathanasiou N., Gennata C., Kiosses V., Markaki L., Zacharia T., Kalligeris A., Chronopoulou K., Karamanidis I., Tsantzali E., Kousoulis G., Simopoulos D., Chatziparasidi M., Doumouliaka M., Panopoulou S., Lazaridou E., Topalidi V., Chatzipetrou V., Vagenas S., Chondros P., Zharkalliu C.
